# Deep learning reveals cuproptosis features assist in predict prognosis and guide immunotherapy in lung adenocarcinoma

**DOI:** 10.3389/fendo.2022.970269

**Published:** 2022-08-19

**Authors:** Gang Li, Qingsong Luo, Xuehai Wang, Fuchun Zeng, Gang Feng, Guowei Che

**Affiliations:** ^1^ Department of Thoracic Surgery, West-China Hospital, Sichuan University, Chengdu, China; ^2^ Department of Thoracic Surgery, Sichuan Provincial People’s Hospital, University of Electronic Science and Technology of China, Chengdu, China; ^3^ Sichuan Translational Medicine Research Hospital, Chinese Academy of Sciences, Chengdu, China

**Keywords:** cuproptosis, deep learning, prognosis, lung adenocarcinoma, risk model

## Abstract

**Background:**

Cuproptosis is a recently found non-apoptotic cell death type that holds promise as an emerging therapeutic modality in lung adenocarcinoma (LUAD) patients who develop resistance to radiotherapy and chemotherapy. However, the Cuproptosis’ role in the onset and progression of LUAD remains unclear.

**Methods:**

Cuproptosis-related genes (CRGs) were identified by a co-expression network approach based on LUAD cell line data from radiotherapy, and a robust risk model was developed using deep learning techniques based on prognostic CRGs and explored the value of deep learning models systematically for clinical applications, functional enrichment analysis, immune infiltration analysis, and genomic variation analysis.

**Results:**

A three-layer artificial neural network risk model was constructed based on 15 independent prognostic radiotherapy-related CRGs. The risk model was observed as a robust independent prognostic factor for LUAD in the training as well as three external validation cohorts. The patients present in the low-risk group were found to have immune “hot” tumors exhibiting anticancer activity, whereas the high-risk group patients had immune “cold” tumors with active metabolism and proliferation. The high-risk group patients were more sensitive to chemotherapy whereas the low-risk group patients were more sensitive to immunotherapy. Genomic variants did not vary considerably among both groups of patients.

**Conclusion:**

Our findings advance the understanding of cuproptosis and offer fresh perspectives on the clinical management and precision therapy of LUAD.

## Introduction

Among malignancies, lung cancer is the most widely known around the globe (1), and a major type of non-small cell lung cancer (NSCLC) is lung adenocarcinoma (LUAD) covering 40% of lung cancers (2, 3). Conventional chemotherapy and radiotherapy remain the main treatment methods for LUAD (4, 5), while patients with advanced LUAD have a higher risk of treatment failure because of the development of treatment resistance (4). Novel treatment modalities have focused on profound changes in the genome, and to date, there are two main therapeutic strategies related to genetic factors; targeted therapy and immunotherapy (6). However, most patients are prone to resistance while receiving targeted therapies and only a minority of patients may benefit from immunotherapy. Therefore, further studies on potential prognostic biomarkers for LUAD are needed to provide better prognostic prediction and tailored treatment.

A novel non-programmed cell death mechanism caused by copper overload, cuproptosis, was recently reported (7). Cuproptosis is regulated by protein lipoylation, and copper binds directly to the lipoylation constituents of the tricarboxylic acid cycle, which leads to the aggregation of lipoacylated proteins and the following loss of iron-sulfur cluster proteins, thus leading to proteotoxic stress and eventually cell death (7). These results indicate that copper ion carriers may serve as an emerging therapeutic modality for cancer, an approach that may be particularly effective in cancers that are naturally resistant to apoptosis (7, 8), and by exploiting the unique action of this metal, a novel approach to killing cancer cells could be found. Despite the good efficacy of conventional treatments in LUAD, a large proportion of patients still experience resistance to radiotherapy or drugs. Therefore, understanding cuproptosis is expected to provide accurate prognosis prediction for LUAD patients and guidance for targeted therapy and immunotherapy for patients who show resistance to treatment.

In our study, we systematically evaluated cuproptosis-related genes (CRGs) in patients with LUAD after radiation therapy and constructed and validated a new cuproptosis-related risk model. The risk model was an independent prognostic factor for overall survival (OS) in patients with LUAD in the training set as well as multiple external validation sets. Additionally, the high-risk group patients had immunosuppressed, proliferatively active “cold” tumors, and the low-risk group patients had anti-tumor-active “hot” tumors. Finally, we predicted that patients in the low-risk group would show increased sensitivity to immunotherapy, whereas patients in the high-risk group would show more sensitivity to chemotherapy.

## Methods

### Data processing

The dataset GSE42172 containing six paired normal A549 lung cancer cell lines and 6 radiation treated A549 lung cancer cell lines were used to explore CRGs before and after radiotherapy. We retrieved TCGA-LUAD patient data from the TCGA database (https://cancergenome.nih.gov/) *via* the GDC API, including transcriptome RNA-Seq data, and Mutect2 platform mutation data, Human Methylation 45 methylation data, copy number variation (CNV) data as well as associated clinical data. A total of 492 LUAD samples were collected after excluding patients that lost to follow-up or lacked clinical information. The primitive FPKM sequencing data were normalized by TPM and used as a training cohort. Three mature LUAD cohorts were taken from GEO (http://www.ncbi.nlm.nih.gov/geo/): GSE30219, GSE42127, and GSE72094, from chip platforms GPL570, GPL6884, and GPL15048, respectively. The three datasets were normalized by log2 and then served as external validation cohorts. In addition, we collected the publicly available established immunotherapy cohorts Imvigor210 and GSE135222, comprising 298 patients with uroepithelial cancer and 27 patients with NSCLC treated with anti-PD-1, respectively.

Finally, single-cell sequencing data were collected for GSE131907, containing 58 sequencing cases from 44 patients. Among them, we selected normal lung tissue, early stage, advanced stage, and brain metastasis lung tissue for further analysis, containing 29 samples in total.

### Identification of the candidate cuproptosis-related genes

Eight cuproptosis genes were collected from Tsvetkov et al. (7), detailed gene list was provided in **Table S1**. For ssGSEA analysis, we employed the R package “gsva” to generate cuproptosis scores, and for multiscale embedded gene co-expression network analysis we used an R package called MEGENA. It shows higher performance than co-expression network construction and is not limited by sample size. Genes with standard deviation > 0.9 were selected for MEGENA analysis, and planar filtered network (PFN) was measured following the gene expression correlation. We applied the multiscale clustering strategy to construct gene networks having modules or interconnected subnetworks, and module characteristic genes were calculated using the module Eigengenes R function to calculate the correlation of modules with cuproptosis scores and to identify the most relevant modules. The genes contained in the most relevant modules were considered radiotherapy-related CRGs and used for subsequent analysis.

### Single-cell data analysis

We employed the R package “Seurat” for processing the scRNA-seq data. Specifically, cells with “min.cells < 3” and “min.features < 200” were excluded. After filtering cells with mitochondrial sequencing counts > 20% and nFeature_RNA > 7000, a total of 58812 cells were retained for subsequent analysis. The dataset was subsequently normalized using the NormalizeData and ScaleData functions of Seurat. Cell types were identified according to the cell annotations provided in the original article. The scanpy in Python was used for the visualization of model genes.

In addition, to reveal changes in cell clusters during tumor progression, we used the R package “monocle” for single-cell trajectory analysis (9). According to the following parameters: num_cells_expressed >= 10, min_expr = 0.1, variable genes of top 1500 were used to identify single-cell developmental trajectory, monocle was used to visualize cell developmental trajectories and dynamic expression of model genes.

### Building and validating predictive deep learning models

We use the R package “survivalmodels” to build an artificial neural network (ANN) model and the “mlr3” package for parameter tuning. First, we train the model in the TCGA dataset, the input layer is the expression of CRGs, the activation function of the hidden layer is ReLU, and the loss function is the negative log partial likelihood under the Cox PH model. Five pieces of cross-validation and dropout parameters were adopted to avoid overfitting, and the early stopping strategy is also utilized for the regularization purpose. The best ANN model is selected based on the C-index and the predicted risk values of individual patients are generated by Cox regression. For evaluating the risk scores’ predictive ability in the training as well as validation sets, and for measuring the consistency C-index we utilized the “survcomp” R package, a larger C-index indicated the model’s higher predictive accuracy and power (10). The median Risk score was used for classifying the patients into high- and low-risk groups, and the prognostic value of the risk model was investigated systematically by Km survival curves, univariate and multivariate Cox regression, and time-dependent ROC curves.

### Gene enrichment and immune infiltration analysis

To study the pathway activity of the samples we did ssGSEA analysis by the R package “gsva”. Gene markers were collected from previously published literature (11–14) and detailed gene markers are demonstrated in **Table S2**. GSEA analysis was also carried out for studying the KEGG pathway variations in high and low CuRS groups and screened for significant pathways by p < 0.05.

Using the R package “CIBERSORT”, we did the estimation of the proportion of immune cell infiltration in tumor samples, which estimates the degree of infiltration of 22 immune cell types (15). The Estimate algorithm was employed to assess the samples’ immune activity and tumor purity (16). Finally, using the study of Thorsson et al, samples of indel neoantigens and SNV neoantigens were also obtained (17). The immunophenotypic score (IPS) of the samples was calculated based on previous studies, with higher IPS indicating stronger immune activity of the samples (18).

### Dissecting the genomic variation landscape between the two subgroups

For comparing the variations in mutation burden in both risk groups, we processed the mutation data using the “maftools” R package, first calculating the total number of mutations in the samples, then identifying genes with a minimum number of mutations > 50, the variations in mutation frequencies of both risk groups were compared using chi-square tests, and visualizing them using maftools. CNV data were processed through Gistic 2.0 on the Genepattern website, significantly amplified and missing chromosomal segments were identified, and CNV summary maps were visualized using the R package Circos.

### Potential small molecule drugs and chemotherapy sensitivity prediction

The CTRP2.0 and PRISM databases were used to predict potentially sensitive small molecule drugs containing sensitivity data for small molecule compounds in cancer cell lines (CCL), CCL expression data from the Cancer Cell Line Encyclopedia (CCLE) project were taken from (https://portals.broadinstitute.org/ccle/), and a ridge regression model was made as per the CCL expression data from the CTRP2.0 and PRISM databases and TCGA expression data using the built-in ridge regression function of the pRRophetic package for predicting the sensitivity of various risk subgroups to small molecule compounds in the TCGA data set. As a standard value for evaluating drug sensitivity, lower AUC values show better drug sensitivity. We also introduced the Genomics of Drug Sensitivity to Cancer (GDSC) database (www.cancerRxgene.org) for predicting differences in the sensitivity of five common lung cancer chemotherapeutic agents in high- and low-risk samples. Moreover, genes that were expressed differentially in the high-risk and low-risk group samples are potential therapeutic targets. Therefore, we used the online database ConnectivityMap (https://clue.io/) to detect potential drugs targeting these genes and elucidate the corresponding mechanisms of action.

### Predicting immunotherapy affect rates

The response of patients to anti-PD1 and anti-CTLA4 therapies was predicted for all cohorts using the TIDE algorithm (http://tide.dfci.harvard.edu) (19). Subsequently, we used the unsupervised subclass mapping (https://cloud.genepattern.org/gp/) and forecast patients’ response to anti-PD-1 and anti-CTLA-4 treatment by transcriptome expression patterns, to predict the response of both high risk and low risk groups to immunotherapy. Finally, we tested the risk model’s predictive efficacy in the immunotherapy cohort Imvigor210.

### Bioinformatics and statistical analysis

The software R 4.1.0 performed all statistical analyses and was used in making all the graphs. Differential genes were obtained by Limma package analysis, and genes with p.adjust < 0.05 and |Log2FC| > 1 were considered to be significantly different. The Kruskal-Wallis test was employed for comparing among multiple groups, and the Wilcoxon test was utilized for comparison between two groups. Variations in proportions were compared by the chi-square test. For each data set, survival curves for subgroups were produced with the help of Kaplan-Meier plotters. The log-rank test determined if the differences were significant. Two-tailed p < 0.05 was considered significant.

## Results

### Identification of radiotherapy-associated cuproptosis features

**Figure 1:**
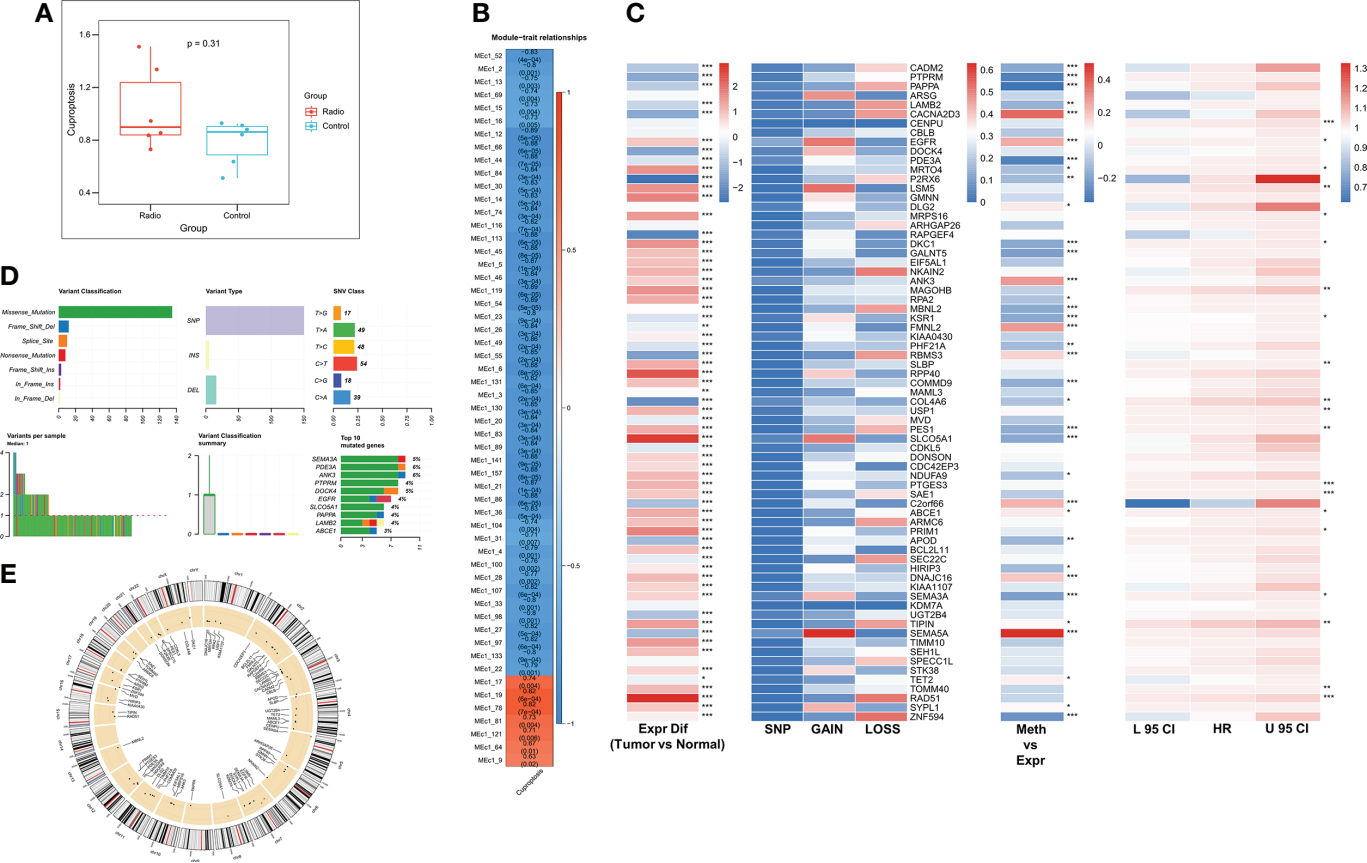
Identification of radiotherapy-associated CRGs. **(A)** Box plot showing Cuproptosis pathway activity before and after radiotherapy. **(B)** Identification of the most relevant gene modules for Cuproptosis. **(C)** Heat map showing genomic changes and hazard ratios of CRGs in TCGA-LUAD. From left to right were respectively: expression differences of CRGs in tumor and normal samples, frequency of mutations and copy number variants of CRGs, correlation of DNA methylation modifications and expression of CRGs, and univariate Cox regression analysis showing risk ratios of CRGs. *p < 0.05, **p < 0.01, ***p < 0.001. **(D)** Summary of mutational events in CRGs in TCGA-LUAD. **(E)** Circle diagram demonstrating the CNV events of CRGs on chromosomes.

We first assessed the altered cuproptosis pathway activity due to radiotherapy using ssGSEA, which showed a trend of increased cuproptosis pathway activity after radiotherapy (P = 0.31, **Figure 1A**). Next, to explore the genes associated with the altered cuproptosis pathway, we performed MEGENA analysis using transcriptome and cuproptosis pathway activity. 55 modules were identified, of which module 19 and its submodule 78 were most associated with the cuproptosis pathway (cor = 0.82, p < 0.001, **Figure 1B**), and both modules contained a total of 71 CRGs for subsequent analysis. We next analyzed the multi-omics profiles of CRGs in TCGA-LUAD (**Figure 1C**), most CRGs were elevated in tumor patients, and we observed a low mutation frequency and CNV frequency of CRGs. However, most CRGs were significantly negatively correlated with their methylation levels, suggesting that CRGs are mainly regulated by methylation in LUAD. Univariate Cox regression analysis revealed that 21 CRGs were unfavorable prognostic factors for OS in LUAD patients. **Figure 1D** summarizes the mutation profile of CRGs in LUAD and shows that SEMA3A, ANK3, and PDE3A are the three CRGs with the highest mutation frequencies. **Figure 1E** shows the CNV mapping of CRGs on chromosomes.

### The single-cell landscape of CRGs

**Figure 2:**
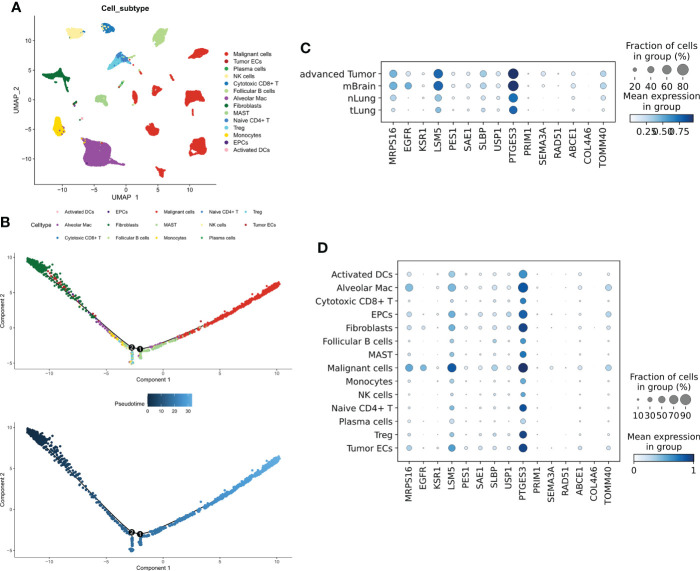
Single-cell transcriptional map of CRGs. **(A)** Umap plots of 14 cell subtypes. **(B)** Quasi sequential trajectories of 14 cell subtypes, top cell trajectories; bottom: Pseudotime trajectories. **(C)** Expression profiles of 15 prognostic CRGs in different tissues. **(D)** Expression profiles of 15 prognostic CRGs in different cell types.

To evaluate the distribution of CRGs in different cells, we analyzed the single-cell dataset GSE131907. A total of 14 types of cells (**Figure 2A**) were identified. The Quasi-time series analysis showed that tumor epithelial cells and plasma cells were distributed at the beginning of the time trajectory, immune cells at the middle of the trajectory, while Malignant cells at the end of the trajectory (**Figure 2B**). 21 prognostic CRGs had 15 intersections in the training cohort and three validation cohorts, afterward, we analyzed the distribution of these 15 prognostic CRGs. The results showed that all prognostic CRGs were elevated in advanced LUAD samples as well as in LUAD samples with brain metastases; in particular, EGFR was more expressed in samples with brain metastases, while PTGES3 was significantly elevated in all tumor samples used (**Figure 2C**). For different cell types, prognostic CRGs expression was increased mainly in Malignant cells, while it was decreased in both immune cells (**Figure 2D**).

### Training and building ANN models

**Figure 3:**
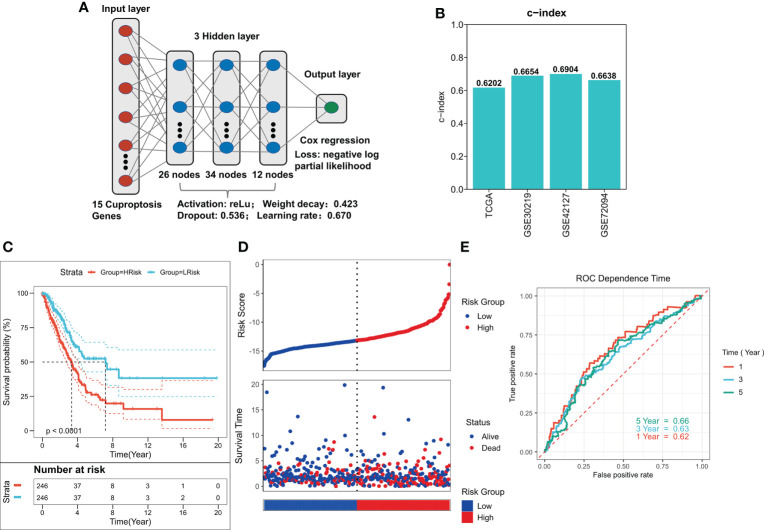
Construction of deep learning model related to CRGs. **(A)** Schematic diagram of the optimum ANN model. **(B)** C-INDEX of the optimum ANN model in the TCGA and GEO cohorts. **(C)** KM survival curves of the high-risk and low-risk groups in the TCGA cohort. **(D)** Survival status of patients in the TCGA cohort. **(E)** ROC curves of risk score at 1, 3, 5, and 8 years in the TCGA cohort.

To train the best ANN model, we trained the model using a random search for 1000 iterations, and the final optimum model is shown in **Figure 3A**. The model has a satisfactory C-index in all four cohorts (C-index > 0.60, **Figure 3B**). Predicted risk scores were generated as per the COX regression, and high- and low-risk patients were distinguished as per the median risk score. A significantly worse prognosis in high-risk patients was observed after survival analysis (P < 0.0001, **Figure 3C**). Survival time was also considerably shorter in the three external validation cohorts of the high-risk group (P < 0.05, **Figures S1A–C**). The risk plot showed a significantly worse survival status in the high-risk group (**Figure 3D**). We saw identical outcomes in the three external validation cohorts (**Figures S1D–F**). ROC analysis demonstrated that the ANN model had the respective AUCs of 0.62, 0.63, and 0.66 at 1, 3, and 5 years (**Figure 3E**), and the ANN model also had satisfactory predictive power in the three external validation cohorts (AUC > 0.6, **Figures S1G–I**).

### Predictive independence of risk models

**Figure 4:**
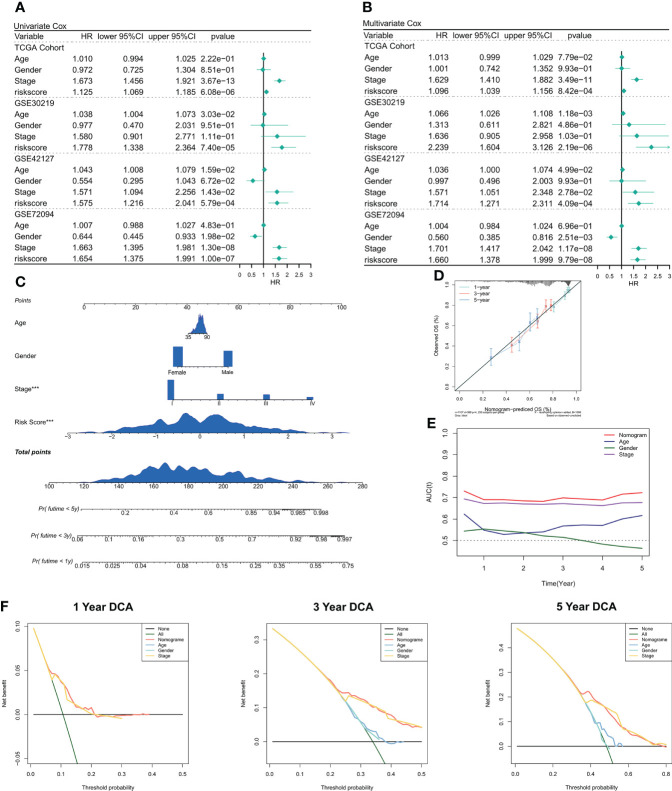
Validation of deep learning models related to CRGs. **(A)** Univariate COX regression of OS in TCGA and GEO datasets. **(B)** Multivariate COX regression of OS in TCGA and GEO datasets. **(C)** Nomogram based on deep learning model of CRGs. **(D)** Calibration curves of Nomogram at 1, 3, and 5 years. **(E)** Nomogram and tROC curves of clinical characteristics. **(F)** DCA curves of Nomogram at 1 year, 3 years, and 5 years.

We used univariate and multivariate Cox regression to assess the relationship between risk scores and patient clinical characteristics and prognosis. In both the training and validation sets, univariate Cox regression served as an independent predictive indicator for risk scores (P < 0.001, **Figure 4A**). After correction for biases such as other clinical traits, multivariate Cox regression showed that risk scores continued to be an independent predictor of OS in both the training and validation cohorts (P < 0.001, **Figure 4B**). Furthermore, as per the subgroup analysis, the risk score remained a reliable prognostic factor in different clinical subgroups (P < 0.05, **Figure S2**). Therefore, risk scores can be a reliable prognostic marker for patients with LUAD. We then created the Nomogram to better quantify the risk assessment of individual LUAD patients (**Figure 4C**). Nomogram correction curves showed good stability and accuracy of the Nomogram model at 1, 3, and 5 years (**Figure 4D**). tROC analysis showed that the Nomogram model was the best predictor at 5 years compared to clinical characteristics (**Figure 4C**). predictor (**Figure 4E**). We then performed a DCA for evaluating the decision benefit of the Nomogram model, and the outcomes revealed that the Nomogram was suitable for risk assessment of patients with LUAD at 1, 3, and 5 years (**Figure 4F**).

### Functional enrichment analysis of risk models

**Figure 5:**
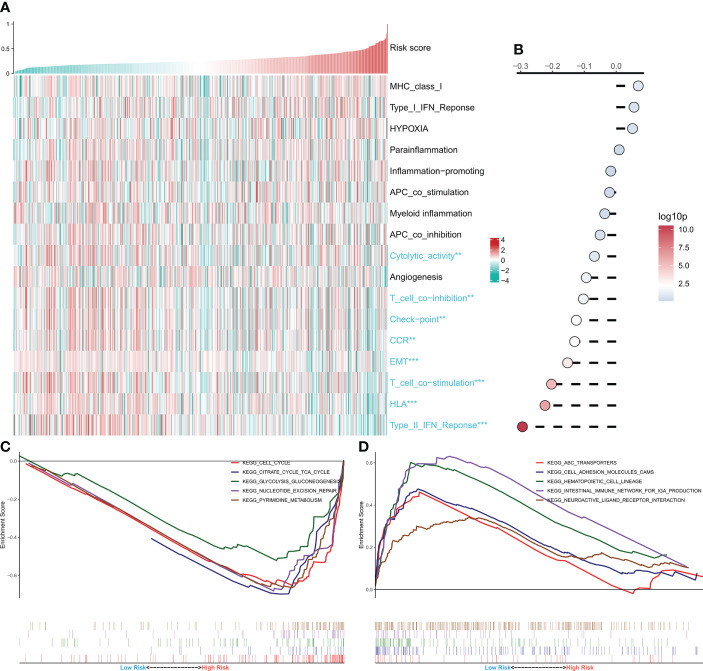
Functional analysis of CRGs-related deep learning models. **(A)** Heat map showing the relationship between Risk score and biological pathway live. **(B)** Correlation analysis of Risk score and biological pathways. **(C)** GSEA enrichment map showing the 5 pathways of interest in the high-risk group. **(D)** GSEA enrichment plot showing the 5 pathways of interest in the low-risk group.

Afterward, the link between the risk model and several typical biological pathways was evaluated. The heat map depicts the link between risk score and biological pathway activity (**Figure 5A**), and the link between risk score and the corresponding biological pathway is shown on the right side of the heat map (**Figure 5B**). The results showed a negative correlation between most of the immunoreactive pathways and the risk score, especially the HLA and Type 2 IFN pathways. Notably, the activity of epithelial-mesenchymal transition was significantly negatively correlated with the risk score and increased in the low-risk group. GSEA analysis showed increased activity of cell cycle, tricarboxylic acid cycle, and glycolysis pathways in the high-risk group (**Figure 5C**), and increased activity of cell adhesion molecules cams and ABC transport pathways in the low-risk group (**Figure 5D**). These outcomes demonstrate that the tumor metabolism and proliferation were active in the high-risk group, while the immune pathway activity was increased in the low-risk group.

### Immune infiltration analysis of the risk model

**Figure 6:**
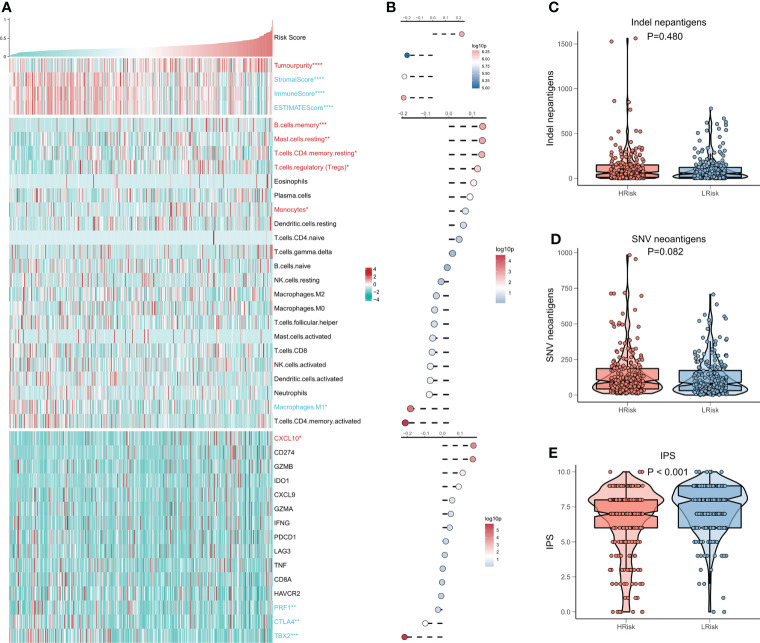
CRGs correlation deep learning model for immune infiltration analysis. **(A)** Heat map displaying the relationship of Risk score, Estimate score, immune cell infiltration abundance, and immune checkpoint table **(B)** From top to bottom: correlation analysis of Risk score and Estimate score, immune cell infiltration abundance, and immune checkpoint expression. **(C)** Box plot demonstrating the difference of indel neoantigens among both risk groups. **(D)** Box plot showing the difference of SNV neoantigens between the high-risk and low-risk groups. **(E)** Box plot demonstrating the difference in IPS among the high- and low-risk groups.

We then evaluated the association between the risk model and the tumor immune microenvironment. The heat map shows the relationship between the risk score and the Estimate Score, the abundance of immune infiltrating cells, and the typical immune checkpoints (**Figure 6A**), and the risk score with the corresponding correlation analysis is shown on the right side of the heat map (**Figure 6B**). The outcomes show a significant increase in tumor purity and Tregs activity in the high-risk group, and notably, a significant increase in memory immune cells and CXCL10 in the high-risk group. In contrast, M1 macrophage activity, as well as three immune checkpoints (PRF1, CTLA4, TBX2), were elevated in the low-risk group. We then examined the number of neoantigens in the high- and low-risk subgroups and showed no considerable difference in the number of Indel neoantigens and SNV neoantigens between the two groups, but there was an increasing trend in the high-risk group (P > 0.05, **Figures 6C, D**). Finally, we found that IPS was higher in the low-risk group in comparison to the high-risk group (P < 0.001, **Figure 6E**). In conclusion, these findings demonstrate that the low-risk group patients have more active antitumor immunity, while the high-risk group patients have a suppressed immune microenvironment.

### Correlation between risk models and genomic variants

**Figure 7:**
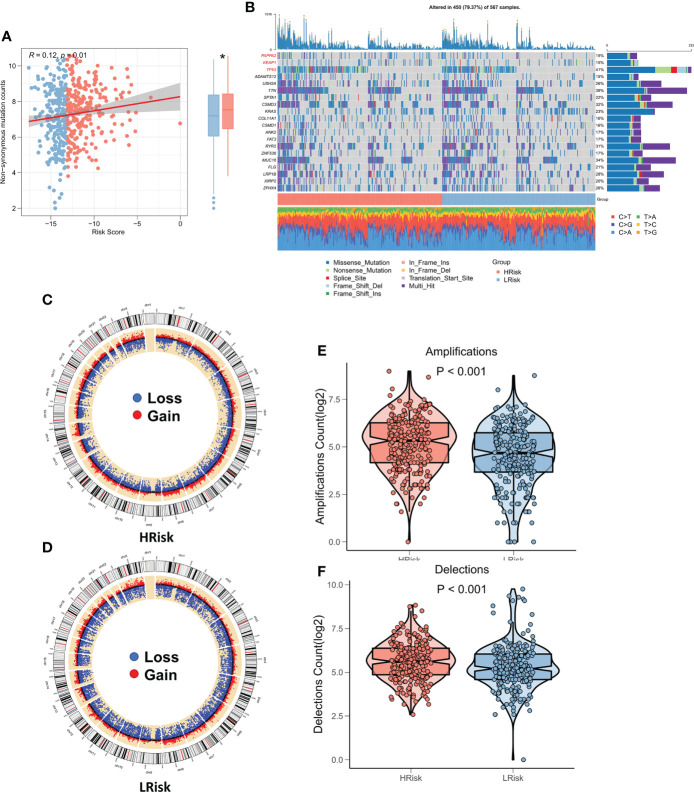
Genomic variation landscape of CRGs-related deep learning models. **(A)** Box plot showing the difference in TMB among the two risk groups. **(B)** Oncoplot of significantly mutated genes between the two risk groups. **(C)** The loop diagram shows the CNV landscape in the high-risk group. **(D)** The loop diagram shows the CNV landscape in the low-risk group. **(E)** Box plots represent the variation in chromosome amplification numbers in the high-risk and low-risk groups. **(F)** Box plots display the variation in the number of chromosome deletions among the two risk groups. *P < 0.05.

Several recent studies have shown that genomic variation is associated with immunotherapy response, for example, more tumor mutational load (TMB) may generate more peptides recognizable by the immune system as potential neoantigens, and antigens containing mutant peptides can activate the immune system and enhance antitumor immunity when recognized (20–22). We studied the correlation between TMB and risk score, and the findings showed that TMB was substantially higher in the high-risk group and was significantly correlated with risk score (**Figure 7A**). The waterfall plot displays the mutation landscape of the top 20 high-frequency mutated genes in the high and low subgroups, with PAPPA2, KEAP1, and TP53 having significantly increased mutation numbers in the high-risk group (**Figure 7B**). The circle plot displays the CNV landscape on the chromosomes of patients in the high-risk and low-risk groups, with the high-risk group patients having more CNV events (**Figures 7C, D**). Specifically, the number of chromosome amplification and chromosome deletions were higher in the high-risk group patients in comparison to the low-risk group patients (P < 0.001, **Figures 7E, F**).

### Risk models can guide clinical decision making

**Figure 8:**
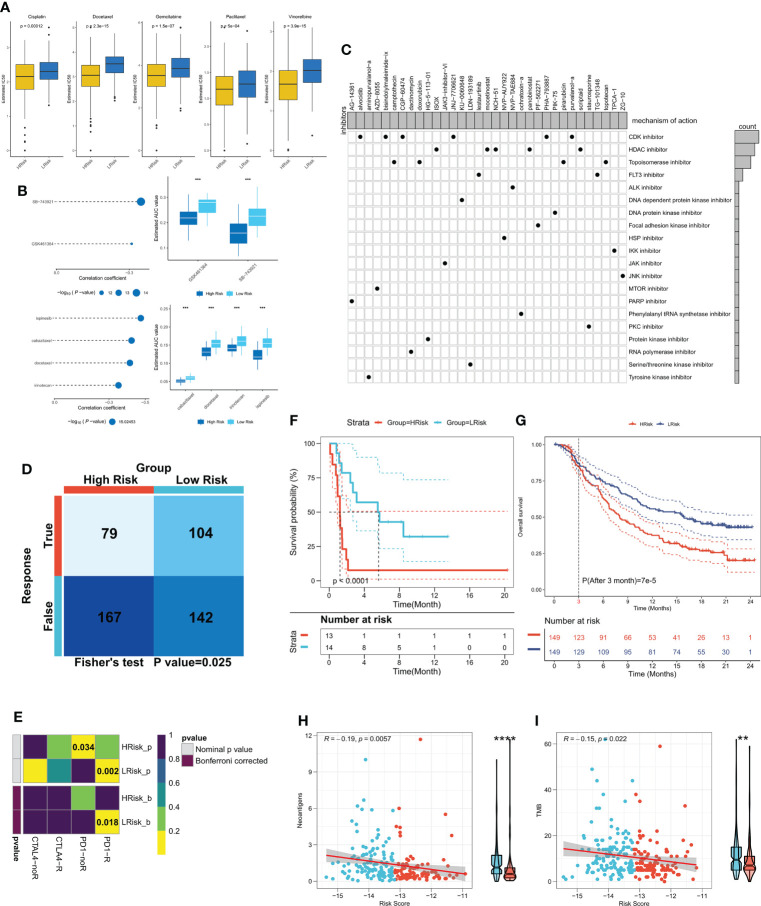
CRGs-related deep learning model in guidance of clinical treatment decisions. **(A)** Box plot showing the predicted IC50 values of five commonly used drugs in the high-risk and low-risk groups. **(B)** Spearman correlation analysis and differential drug analysis of small molecule compounds and Risk Score. Top: CTRP database; bottom: PRISM database. **(C)** Oncoplot represents the identified small molecule compounds, with the horizontal axis representing the small molecule inhibitor name and the vertical axis representing the biological pathway targeted by the small molecule inhibitor. **(D)** The TIDE algorithm was used for predicting the response of the two risk groups to immunotherapy. **(E)** Subclass mapping was employed to predict the sensitivity of patients in both risk groups to PD1 and CTLA4 treatments. **(F)** KM survival curves for both risk groups in the NSCLS immunotherapy cohort. **(G)** KM survival curves for the two risk groups in the IMvigor210 cohort. **(H)** The correlation between risk score and neoantigens in the IMvigor210 cohort; **(I)** The correlation between risk score and TMB in the IMvigor210 cohort. **P < 0.01, ***P < 0.001, ****P < 0.0001.

Differences between the immune and metabolic activities of the high- and low-risk group patients may predict different outcomes in terms of treatment benefits, so we first analyzed the IC50 differences between the two patient groups for five common lung cancer drugs (cisplatin, docetaxel, gemcitabine, paclitaxel, and vincristine). The outcomes revealed that the IC50 of all five drugs was considerably lower in the high-risk group patients in comparison to the low-risk group patients (**Figure 8A**), and the same results were observed in three external validation cohorts (P < 0.05, **Figures S3A–C**), which indicated higher sensitivity of the high-risk group patients to chemotherapy. Considering that the high-risk group patients were more sensitive to chemotherapy, we predicted possible small molecule compounds using the CTRP and PRISM databases. The results are shown in **Figure 8B**. The CTRP database shows that the high-risk group patients may benefit from GSK461364 and SB-743921. Specifically, the Prism database also showed that the high-risk group patients may benefit from docetaxel, suggesting that docetaxel may be a sensitive drug for cuproptosis. We then submitted top150 up-and down-regulated genes between the high- and low-risk subgroups to the cmap database, and a total of 33 possible targeted small molecule drugs were found (**Figure 8C**). Subsequently, the TIDE algorithm revealed higher sensitivity of the low-risk group patients to immunotherapy (P < 0.05, **Figure 8D**), subclass mapping also demonstrated that the patients with low-risk score were more sensitive to immunotherapy (P<0.05, **Figure 8E**), a result confirmed in the external validation cohort (P < 0.05, **Figures S3D–F**). The same result was observed in the validation cohort (P < 0.05, **Figures S3G–I**). Finally, we found significantly longer survival times in the immunotherapy cohort for NSCLC in patients with low-risk scores (P < 0.0001, **Figure 8F**). In a large immunotherapy cohort with long-term follow-up, taking into account the effect of treatment delay, we found that patients with low-risk scores survived longer in comparison to patients with high-risk scores at three months (P < 0.0001, **Figure 8G**). Moreover, compared with the high-risk group, patients in the low-risk group had more TMB and neoantigens, suggesting that low risk score is correlated with a high response to immunotherapy (P<0.01, **Figures 8H, I**). In conclusion, these results suggest that the high-risk group patients are more responsive to chemotherapy, whereas patients in the low-risk group are more responsive to immunotherapy.

## Discussion

LUAD is the most widely known type of lung cancer and among the major causes of death that occur as a result of cancer. Due to the significant heterogeneity of LUAD, patients with advanced LUAD often show resistance to conventional treatment modalities, especially radiotherapy (4). Therefore, it is difficult to predict prognosis and develop individualized treatment strategies timely. Cuproptosis is a recently reported modality of cell death that could be an emerging treatment modality for cancer, and this approach may be particularly effective in cancers that are naturally resistant to apoptosis (7, 8). Therefore, this study focused on CRGs after LUAD radiotherapy and developed a robust prognostic model that could be utilized as an independent prognostic factor for patients with LUAD. Moreover, this model can distinguish between “hot” tumors with active antitumor immunity and “cold” tumors with active metabolism and proliferation and can inform the development of chemotherapy and immunotherapy regimens.

Cell death is strongly associated with cancer progression, metastasis, and treatment response, and inhibition of cell death increases tumor metastasis and resistance of malignant cells to chemotherapy (23, 24). Induction of cell death mechanisms rather than apoptosis has emerged as a new cancer treatment strategy, as most tumors are innately resistant to apoptosis (25). The development of bioinformatics technologies has taken a leading edge in analyzing complex genomic changes, and there have been numerous studies demonstrating the potential of transcriptome analysis in predicting lung cancer patient prognosis (26–28). We focused for the first time on CRGs in LUAD patients after radiotherapy in an attempt to provide a prognostic prediction for radiotherapy-resistant LUAD patients, and we found that CRGs were significant prognostic factors in LUAD, and advanced ANN models based on CRGs showed excellent predictive efficacy in both the training and external validation datasets, with OS reduced greatly in high-risk patients.

In this study, we confirmed that biological pathways differed significantly between patients in different Cuproptosis subgroups, and we found significantly increased activity of cell cycle pathways, glycolytic pathways, and tricarboxylic acid cycle pathways in the high-risk group patients. It was demonstrated that excessive cell cycle hyperactivity is closely associated with cancer cell proliferation (29, 30), and proliferating tumor cells are heavily dependent on glycolysis and the tricarboxylic acid cycle for energy supply (31, 32). Conversely, the low-risk group patients had increased activity of immune-related pathways, particularly the HLA and Type 2 IFN pathways. To gain more insight into the differences in immune activity, we then studied the variations in the immune microenvironment of the two subgroups. The Estimate algorithm showed a higher tumor purity in the high-risk group and higher immune scores in the low-risk group. In addition, M1 macrophage abundance, PRF1, CTLA4, and TBX2 were elevated in the low-risk group. According to studies, M1 macrophages are central anti-tumor cells and can act as therapeutic vectors (33, 34). The activity of memory immune cells was increased in the high-risk group; however, the increased Tregs may have contributed to the immunosuppressive microenvironment in the high-risk group patients (35, 36). Furthermore, CXCL10 is upregulated in the high-risk group and may be able to act as a molecular target for activating antitumor immunity in patients in the high-risk group (37).

As per the reports, TMB can be a biomarker of immunotherapeutic response, and higher TMB suggests better immunotherapeutic response (38, 39). However, the predictive efficacy of TMB often shows heterogeneity across cancer types (40). We discovered that the TMB was greater in the high-risk group of patients. However, as mentioned above, the high-CuRS group patients exhibited lower immunoreactivity, showing that high TMB doesn’t necessarily predict high immunogenicity. Additionally, prior research has demonstrated that TMB is not a perfect predictor of immunotherapy in NSCLC (41, 42), so we propose that Cuproptosis-related risk models can better identify “hot” tumors with an immune activation phenotype and guide immunotherapy.

We predicted that the high-risk group patients would show higher sensitivity to chemotherapy, especially Docetaxel; however, patients with LUAD at advanced stages often develop resistance to chemotherapeutic agents. Immunotherapy is a new therapy for many cancers, like NSCLC, and exploring the patient types that immunotherapy can help with is still a challenge. Further, we evaluated the efficacy of risk models in predicting immunotherapy in patients with LUAD from different perspectives. Patients with higher risk scores were shown to be more responsive to anti-PD1 treatment in TIDE and subclass mapping studies, and this finding was supported by data from an external validation cohort. By analyzing a small cohort of NSCLC patients receiving immunotherapy, we found a significant increase in survival time for patients with low-risk scores. More convincingly, we demonstrated in a large immunotherapy cohort IMvigor210 that patients with low-risk scores survived better in comparison with those having high risk. The current findings showed that the genomic variation of tumor cells often leads to a larger number of tumor-specific mutant peptides, which could be used as neoantigens for the immune system to recognize. The recognition of new antigens is the main factor of clinical immunotherapy activity (43, 44). Therefore, more neoantigens and TMB in the low-risk group may result in the increased response of patients with low risk scores to immunotherapy. In conclusion, we propose that a strategic optimization scheme for Cuproptosis-based chemotherapy and immunotherapy may be effective.

This study has several limitations. First, the data utilized in our work were taken from public databases, like the TCGA and GEO databases. Therefore, it is not possible to assess the quality of the data, and multi-center validation is still needed before applying it to clinical practice, especially for large prospective studies. Second, our data are Bulk-seq data, which only consider inter-patient heterogeneity and not intra-tumor heterogeneity. Finally, additional *in vivo* as well as *in vitro* experiments to explore the specific biological mechanisms of Cuproptosis in LUAD are needed.

In summary, our work presents a novel Cuproptosis-related prognostic model for LUAD that has shown excellent performance in multiple datasets. Functionally, low-risk patients suggest active antitumor immunity and immune activation of “hot” tumors. In addition, we determined that the model could be used for predicting the sensitivity of chemotherapy and immunotherapy in individuals with LUAD. These results advance the understanding of Cuproptosis and the clinical management and precise treatment of LUAD.

## Data availability statement

The original contributions presented in the study are included in the article/**Supplementary Material**. Further inquiries can be directed to the corresponding authors.

## Author contributions

GL drafted the manuscript; QL and XW contributed significantly to analysis and manuscript preparation; FZ and GF performed the data analyses; GC contributed to the conception of the study. All authors contributed to the article and approved the submitted version.

## Acknowledgments

The authors hereby express their gratitude to all participants who supported this study, especially the TCGA and GEO database providers who provided the data for the analysis.

## Conflict of interest

The authors declare that the research was conducted in the absence of any commercial or financial relationships that could be construed as a potential conflict of interest.

## Publisher’s note

All claims expressed in this article are solely those of the authors and do not necessarily represent those of their affiliated organizations, or those of the publisher, the editors and the reviewers. Any product that may be evaluated in this article, or claim that may be made by its manufacturer, is not guaranteed or endorsed by the publisher.

## Supplementary material

Supplementary Figure 1 External validation of CRGs-related deep learning models. **(A)** KM survival curves of the high-risk and low-risk groups in the **(A)** GSE30219, **(B)** GSE42127, and **(C)** GSE72094 cohorts. **(D)** Patients’ survival status in the GSE30219, **(E)** GSE42127, and **(F)** GSE72094 cohorts. **(G)** ROC curves of risk score at 1 year, 3 years, and 5 in the GSE30219, **(H)** GSE42127, and **(I)** GSE72094 cohorts.

Supplementary Figure 2 Subgroup Cox analysis of FRS. Subgroup Cox regression analysis of risk score in **(A)** TCGA cohort, **(B)** GSE30219, **(C)** GSE42127, and **(D)** GSE72094 cohorts.

Supplementary Figure 3 CRGs-related deep learning model for treatment decision. **(A)** Predicted IC50 values of the five commonly used drugs in the two risk groups in the **(A)** GSE30219, **(B)** GSE42127, and **(C)** GSE72094 cohorts. Differences in immunotherapy response predicted by the TIDE algorithm between both risk groups in the **(D)** GSE30219, **(E)** GSE42127, and **(F)** GSE72094 cohorts. Differences in immunotherapy response between both risk groups were predicted by subclass mapping in the **(G)** GSE30219, **(H)** GSE42127, and **(I)** GSE72094 cohorts.
