# Supplementary figures and images for: Deep learning reveals cuproptosis features assist in predict prognosis and guide immunotherapy in lung adenocarcinoma
Source: Front Endocrinol (Lausanne). 2022 Aug 19;13:970269. doi: 10.3389/fendo.2022.970269 (PMC9437348; doi:10.3389/fendo.2022.970269)

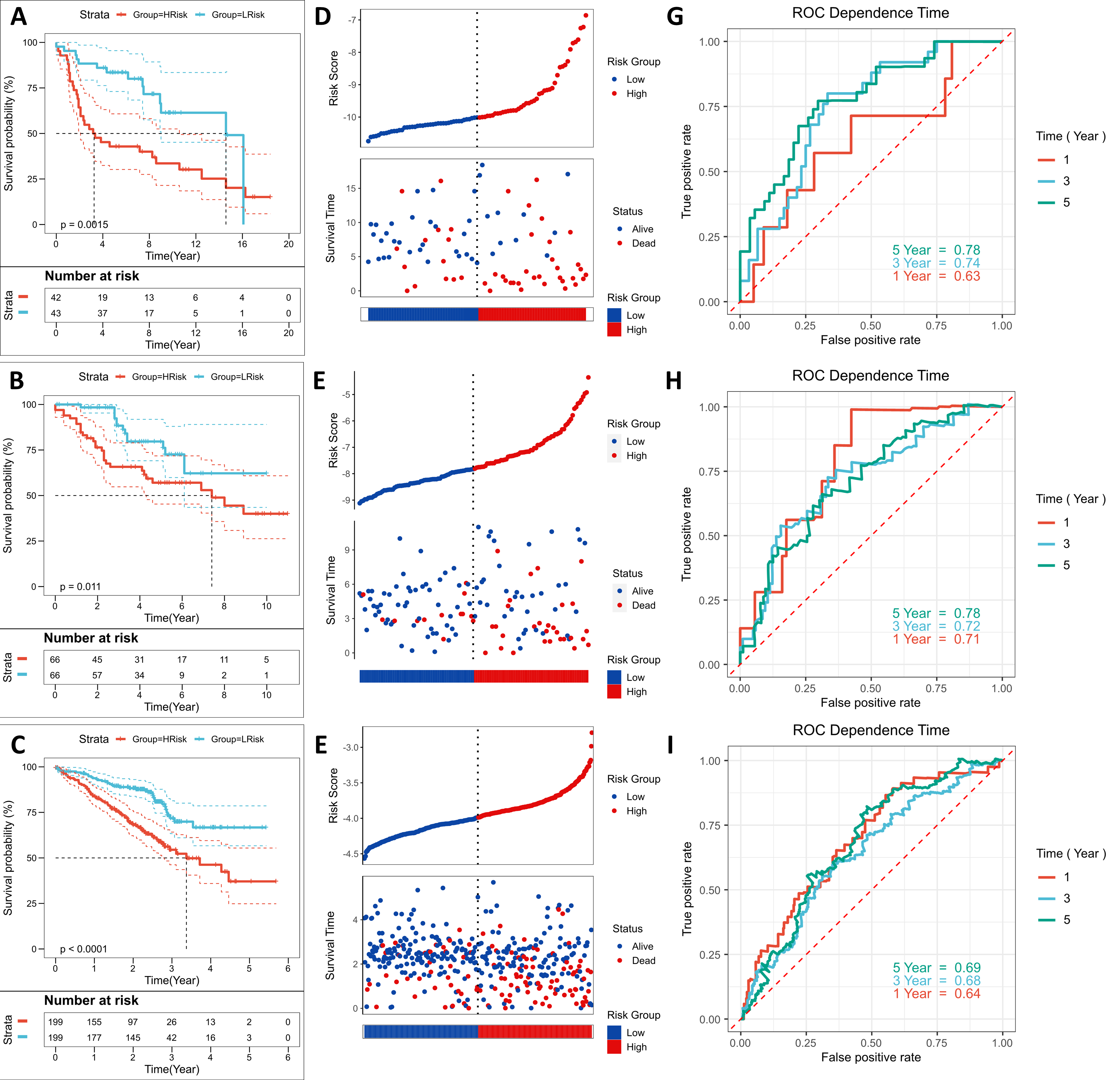

Supplement: Supplementary Figure 1 — External validation of CRGs-related deep learning models. (A) KM survival curves of the high-risk and low-risk groups in the (A) GSE30219, (B) GSE42127, and (C) GSE72094 cohorts. (D) Patients’ survival status in the GSE30219, (E) GSE42127, and (F) GSE72094 cohorts. (G) ROC curves of risk score at 1 year, 3 years, and 5 in the GSE30219, (H) GSE42127, and (I) GSE72094 cohorts. [file Image_1.tif]

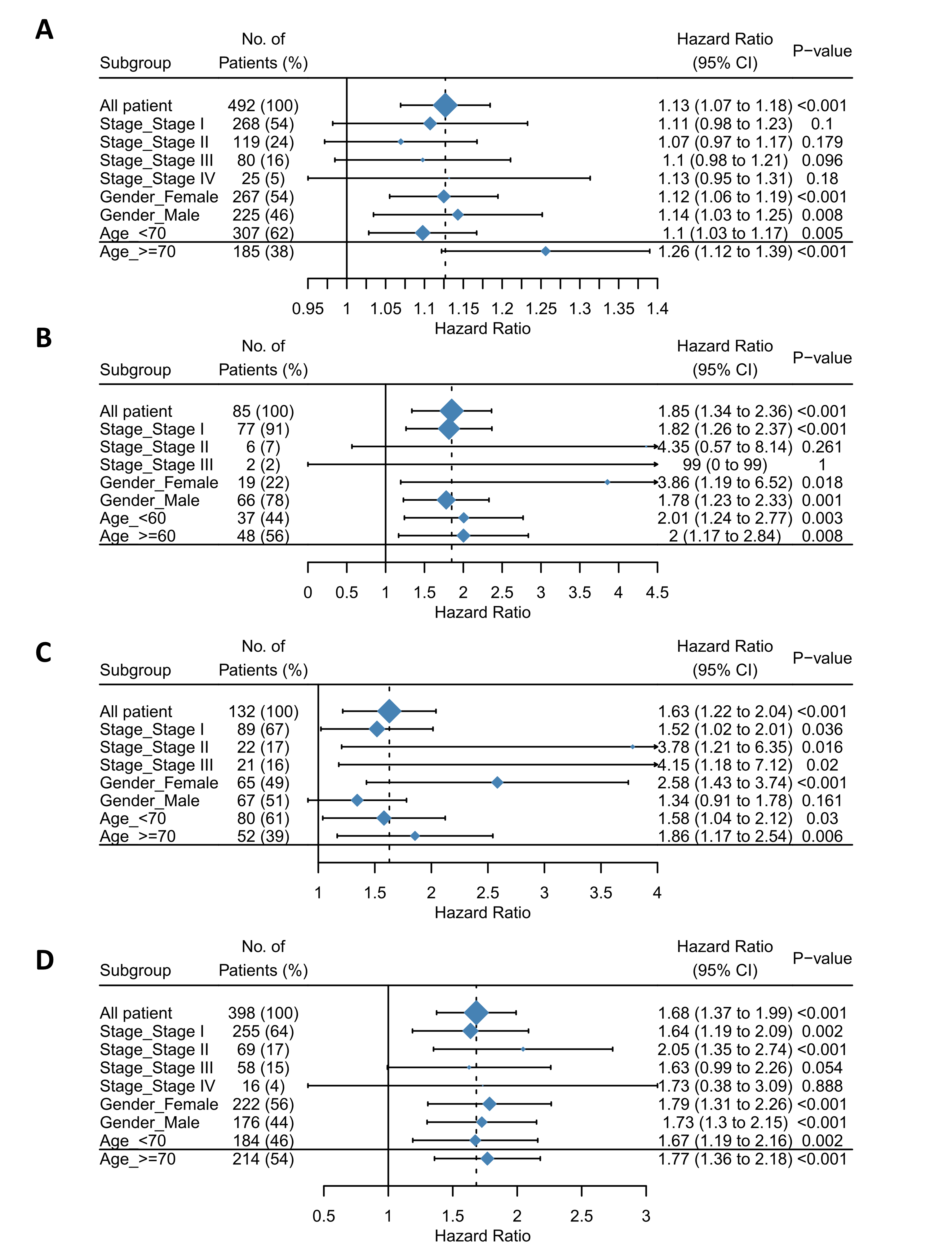

Supplement: Supplementary Figure 2 — Subgroup Cox analysis of FRS. Subgroup Cox regression analysis of risk score in (A) TCGA cohort, (B) GSE30219, (C) GSE42127, and (D) GSE72094 cohorts. [file Image_2.tif]

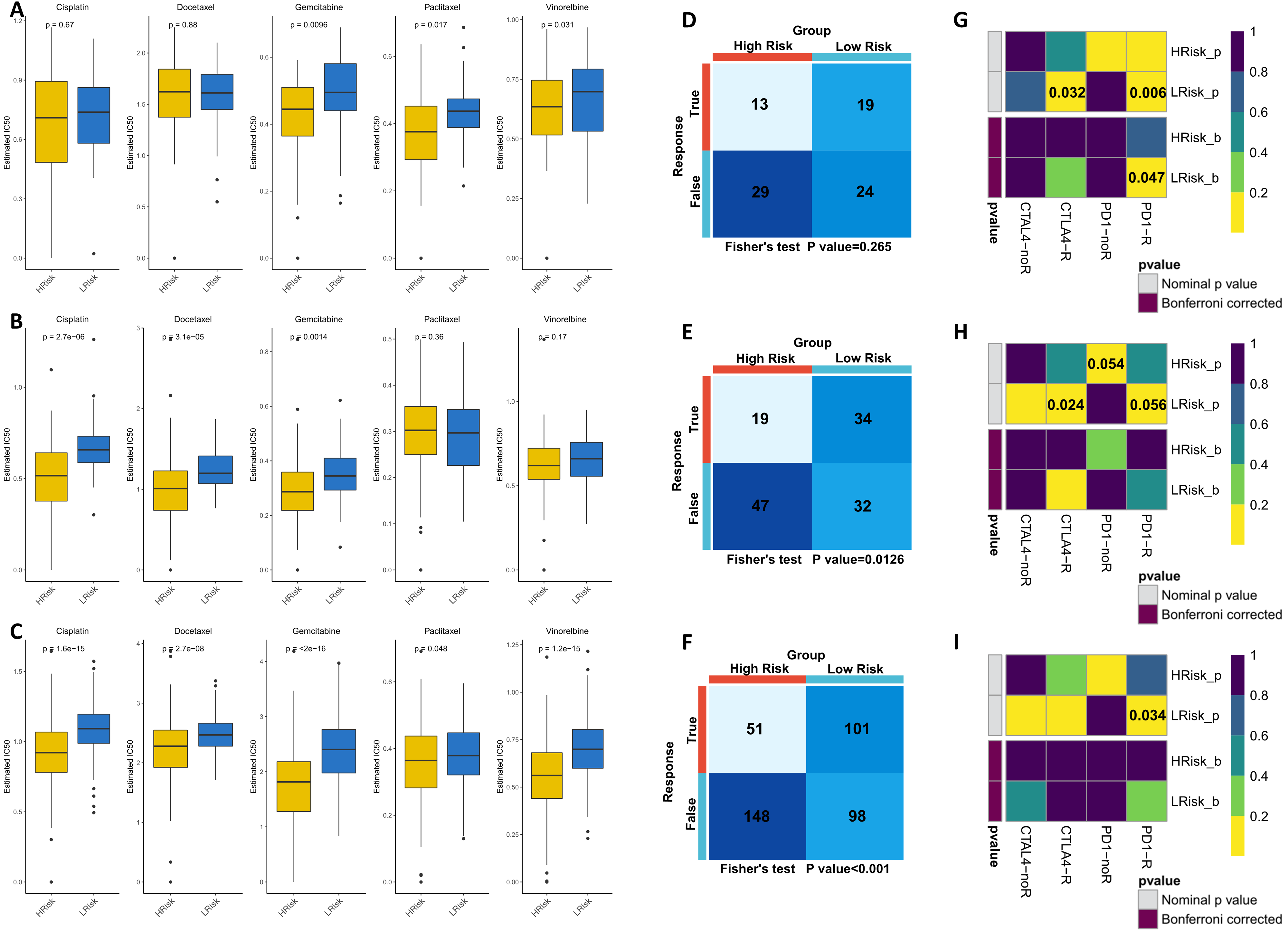

Supplement: Supplementary Figure 3 — CRGs-related deep learning model for treatment decision. (A) Predicted IC50 values of the five commonly used drugs in the two risk groups in the (A) GSE30219, (B) GSE42127, and (C) GSE72094 cohorts. Differences in immunotherapy response predicted by the TIDE algorithm between both risk groups in the (D) GSE30219, (E) GSE42127, and (F) GSE72094 cohorts. Differences in immunotherapy response between both risk groups were predicted by subclass mapping in the (G) GSE30219, (H) GSE42127, and (I) GSE72094 cohorts. [file Image_3.tif]
