# Supplementary material for: Deep learning reveals cuproptosis features assist in predict prognosis and guide immunotherapy in lung adenocarcinoma
Source: Front Endocrinol (Lausanne). 2022 Aug 19;13:970269. doi: 10.3389/fendo.2022.970269 (PMC9437348; doi:10.3389/fendo.2022.970269)
Supplement: Supplementary file 6 [file Table_3.doc]

#### load library ####

library(openxlsx)

# library(stringr)

library(dplyr)

# library(stringr)

# library(DescTools)

library(tidyr)

# library(heaven)

# library(data.table)

library(survival)

# registerDoSEQ()

#### prepare the training and test data sets ####

## load training data

train.gene <- read.delim("data/training/gene-tcga.txt")

train.clinical <- read.delim("data/training/clinical-tcga.txt")

## load test data

test.gene <- read.delim("data/test/gene.txt")

test.clinical <- read.delim("data/test/clinical-icgc.txt")

# write.xlsx(train.gene, file.path(getwd(), "data/train.gene.xlsx"), overwrite = T)

# write.xlsx(train.clinical, file.path(getwd(), "data/train.clinical.xlsx"), overwrite = T)

n <- nrow(train.clinical)

ngene <- nrow(train.gene)

train.clinical$sampleID <- 1:n

train <- cbind(train.clinical, t(train.gene[,-1]))

names(train)[-(1:4)] <- train.gene$Tag

common.gene <- intersect(test.gene$gene_id, train.gene$Tag)

remove.gene <- train.gene$Tag[!train.gene$Tag %in% common.gene]

train <- train[, !(names(train) %in% remove.gene)]

n <- nrow(test.clinical)

ngene <- nrow(test.gene)

test.clinical$sampleID <- 1:n

test <- cbind(test.clinical, t(test.gene[,-1]))

names(test)[-(1:4)] <- test.gene$gene_id

# install.packages(c("ggplot2",

# "mlr3benchmark",

# "mlr3pipelines",

# "mlr3proba",

# "mlr3tuning",

# "mlr3verse",

# "survivalmodels"))

# remotes::install_github("mlr-org/mlr3extralearners")

## L2 regularization: pysurvival.models.semi_parametric.CoxPHModel

library(survivalmodels)

# install_pycox(pip = TRUE, install_torch = TRUE)

# install_keras(pip = TRUE, install_tensorflow = FALSE)

# normalization

data <- as.data.frame(rbind(train[, c(-1, -4)], test[, c(-1, -4)]))

trainindex <- 1: nrow(train)

ngene <- ncol(train) - 4

names(data)[1] <- "time"

data[, c(-1,-2)] <- scale(data)[, c(-1,-2)]

# round(colMeans(data[, c(-1,-2)]))

# apply(data[, c(-1,-2)], 2, sd)

# train.scale <- data[trainindex, ]

# test.scale <- data[-trainindex, ]

# # draft

# n.nodes.1layer <- c(seq(5,45,5),seq(50,150,10))

# CI.1layer.train <- rep(0, length(n.nodes.1layer))

# CI.1layer.test <- rep(0, length(n.nodes.1layer))

# for (i in 1:length(n.nodes.1layer)){

# # dropout, weight_decay, learning_rate

# set.seed(123)

# fit <- deepsurv(data = data[trainindex, ],

# frac = 0.3,

# activation = "relu",

# num_nodes = c(as.integer(n.nodes.1layer[i])),

# dropout = 0.4,

# early_stopping = TRUE,

# epochs = 1500L,

# batch_size = 32L)

# p <- predict(fit, type = "risk", newdata = data[trainindex, ])

# CI.1layer.train[i] <- cindex(risk = p, truth = data[trainindex, "time"])

# p <- predict(fit, type = "risk", newdata = data[-trainindex, ])

# CI.1layer.test[i] <- cindex(risk = p, truth = data[-trainindex, "time"])

# }

library(mlr3)

library(mlr3proba)

library(mlr3verse)

mlr_learners

task <- as_task_surv(data,

id = "genes",

time = "time",

event = "status",

type = "right")

print(task)

head(task$truth())

# kaplan-meier plot

library("mlr3viz")

autoplot(task)

# training and test index

train_set <- trainindex

test_set <- setdiff(seq_len(task$nrow), train_set)

#### tuning hyper-parameters for deepsurv####

# all hyper-parameters:

# https://rdrr.io/github/mlr-org/mlr3extralearners/man/mlr_learners_surv.deepsurv.html

mlr_learners$get("surv.deepsurv")

learner <- lrn(

paste0("surv.", c("deepsurv")),# paste0("surv.", c("deepsurv")),

frac = 0.3,

early_stopping = TRUE,

epochs = 1000,

activation = "relu", # tanh?

optimizer = "adam" # "sgd"?

)

genes <- TaskSurv$new(id = "genes",

backend = data[train_set,],

time = "time",

event = "status",

type = "right")

## create the hyper-parameter search space

library(paradox)

search_space <- ps(

## p_dbl for numeric valued parameters

dropout = p_dbl(lower = 0, upper = 1), # Dropout fraction tuned over [0, 1]

weight_decay = p_dbl(lower = 0, upper = 0.5), # Weight decay over [0, 0.5]

learning_rate = p_dbl(lower = 0, upper = 1), # Learning rate over [0, 1]

## p_int for integer valued parameters

nodes = p_int(lower = 1, upper = 50), # Number of nodes in a layer over {1,...,30}

k = p_int(lower = 1, upper = 4) # Number of hidden layers over {1,...,2}

)

search_space$trafo <- function(x, param_set) {

x$num_nodes = rep(x$nodes, # the number of nodes in a layer

x$k) # the number of layers

x$nodes = x$k = NULL

return(x)

}

# lrns <- mlr_learners$mget(mlr_learners$keys("^surv"))

# names(lrns)

instance <- TuningInstanceSingleCrit$new(

task = genes,

learner = learner,

search_space = search_space,

#resampling = rsmp("holdout"),#rsmp("cv", folds = 3), # 3-fold nested cross-validation

resampling = rsmp("cv", folds = 3),

measure = msr("surv.cindex"),

terminator = trm("evals", n_evals = 500) # 60+ iteration random search

)

tuner <- tnr("random_search")

tuner$optimize(instance)

# weight_decay learning_rate nodes k learner_param_vals x_domain surv.harrell_c

# 0.3611936 0.3127723 46 2 <list[7]> <list[3]> 0.6747061

# dropout weight_decay learning_rate nodes k learner_param_vals x_domain surv.harrell_c

# 0.1138598 0.08566956 0.7322747 47 1 <list[9]> <list[4]> 0.6550695

# instance$result_learner_param_vals

# instance$result_y

# instance$archive

# instance$archive$benchmark_result

# instance$archive$benchmark_result$score(msr("surv.cindex"))

# select the optimal values for hyperparameters

learner$param_set$values = instance$result_learner_param_vals

# train the model using the training data

learner$train(task, row_ids = train_set)

# predict using the test data

res.pred <- learner$predict(task, row_ids = test_set)

res.pred$score()

res <- as.data.table(res.pred)

hist(res$crank)

## create high-risk and low-risk groups according to crank

median(res$crank)

test.clinical$group <- res$crank>=median(res$crank)

library("survival")

library("survminer")

fit <- survfit(Surv(futime, status) ~ group, data = test.clinical)

print(fit)

summary(fit)

ggsurvplot(fit,

pval = TRUE, conf.int = TRUE,

risk.table = TRUE, # Add risk table

risk.table.col = "strata", # Change risk table color by groups

linetype = "strata", # Change line type by groups

surv.median.line = "hv", # Specify median survival

ggtheme = theme_bw(), # Change ggplot2 theme

palette = c("#E7B800", "#2E9FDF"))

# HR under unadjusted Cox PH model

fit.cox <- coxph(Surv(futime, status) ~ group, data = test.clinical)

fit.cox

ggforest(fit.cox)

#### km ####

learner.km = lrn("surv.kaplan")

res.pred.km = learner.km$train(task, row_ids = train_set)$predict(task, row_ids = test_set)

head(as.data.table(res.pred.km))

res.pred.km$score()

#### coxph ####

learner.cox = lrn("surv.coxph")

res.pred.cox = learner.cox$train(task, row_ids = train_set)$predict(task, row_ids = test_set)

head(as.data.table(res.pred.cox))

res.pred.cox$score()

#### appendix: auto tuning - model selection ####

library(mlr3)

library(mlr3proba)

genes <- TaskSurv$new(id = "genes",

backend = train.scale,

time = "time",

event = "status",

type = "right")

## combine in list

tasks <- list(genes)

## create the hyper-parameter search space

library(paradox)

search_space <- ps(

## p_dbl for numeric valued parameters

dropout = p_dbl(lower = 0, upper = 1), # Dropout fraction tuned over [0, 1]

weight_decay = p_dbl(lower = 0, upper = 0.5), # Weight decay over [0, 0.5]

learning_rate = p_dbl(lower = 0, upper = 1), # Learning rate over [0, 1]

## p_int for integer valued parameters

nodes = p_int(lower = 1, upper = 50), # Number of nodes in a layer over {1,...,30}

k = p_int(lower = 1, upper = 4) # Number of hidden layers over {1,...,2}

)

search_space$trafo <- function(x, param_set) {

x$num_nodes = rep(x$nodes, # the number of nodes in a layer

x$k) # the number of layers

x$nodes = x$k = NULL

return(x)

}

# tuning multiple similar learners

library(mlr3tuning)

create_autotuner <- function(learner) {

AutoTuner$new(

learner = learner,

search_space = search_space,

#resampling = rsmp("holdout"),#rsmp("cv", folds = 3), # 3-fold nested cross-validation

resampling = rsmp("cv", folds = 3),

measure = msr("surv.cindex"),

terminator = trm("evals", n_evals = 20), # 60 iteration random search

tuner = tnr("random_search")

)

}

## learners are stored in mlr3extralearners

library(mlr3extralearners)

## load learners

learners <- lrns(

paste0("surv.", c("deepsurv")),# paste0("surv.", c("deepsurv")),

frac = 0.3, early_stopping = TRUE, epochs = 1000, optimizer = "adam" # epochs = 500, optimizer = "sgd", activation = "tanh" "SELU" "ReLU"

)

# apply our function

learners <- lapply(learners, create_autotuner)

## Pre-processing

library(mlr3pipelines)

create_pipeops <- function(learner) {

po("encode") %>>% po("scale") %>>% po("learner", learner)

}

## apply our function

learners <- lapply(learners, create_pipeops)

## benchmark

## select holdout as the resampling strategy

resampling <- rsmp("cv", folds = 3)

# ## add KM and CPH

# learners <- c(learners, lrns(c("surv.kaplan", "surv.coxph")))

design <- benchmark_grid(tasks, learners, resampling)

print(design)

set.seed(123)

bm <- benchmark(design, store_models = T)

## Aggreggate with Harrell's C and Integrated Graf Score

msrs <- msrs(c("surv.cindex"))

mlr3misc::map(as.data.table(bm)$learner, "model")

mlr3misc::unnest(bm$aggregate(params = TRUE), "params")

rr = bm$aggregate()[learner_id == "encode.scale.surv.deepsurv.tuned", resample_result][[2]]

print(rr)

rr$predictions()[[1]]

#### appendix: auto tuning - example ####

library(mlr3)

library(mlr3proba)

## get the `whas` task from mlr3proba

whas <- tsk("whas")

## create our own task from the rats dataset

rats_data <- survival::rats

## convert characters to factors

rats_data$sex <- factor(rats_data$sex, levels = c("f", "m"))

rats <- TaskSurv$new(id = "rats",

backend = rats_data,

time = "time",

event = "status",

type = "right")

## combine in list

tasks <- list(whas, rats)

## create the hyper-parameter search space

library(paradox)

search_space <- ps(

## p_dbl for numeric valued parameters

dropout = p_dbl(lower = 0, upper = 1), # Dropout fraction tuned over [0, 1]

weight_decay = p_dbl(lower = 0, upper = 0.5), # Weight decay over [0, 0.5]

learning_rate = p_dbl(lower = 0, upper = 1), # Learning rate over [0, 1]

## p_int for integer valued parameters

nodes = p_int(lower = 1, upper = 32), # Number of nodes in a layer over {1,...,30}

k = p_int(lower = 1, upper = 4) # Number of hidden layers over {1,...,2}

)

search_space$trafo <- function(x, param_set) {

x$num_nodes = rep(x$nodes, # the number of nodes in a layer

x$k) # the number of layers

x$nodes = x$k = NULL

return(x)

}

# tuning multiple similar learners

library(mlr3tuning)

create_autotuner <- function(learner) {

AutoTuner$new(

learner = learner,

search_space = search_space,

resampling = rsmp("holdout"),#rsmp("cv", folds = 3), # 3-fold nested cross-validation

measure = msr("surv.cindex"),

terminator = trm("evals", n_evals = 2), # 60 iteration random search

tuner = tnr("random_search")

)

}

## learners are stored in mlr3extralearners

library(mlr3extralearners)

## load learners

learners <- lrns(

paste0("surv.", c("deepsurv")),# paste0("surv.", c("deepsurv")),

frac = 0.3, early_stopping = TRUE, epochs = 10, optimizer = "adam" # epochs = 500, optimizer = "sgd", activation = "tanh" "SELU" "ReLU"

)

# apply our function

learners <- lapply(learners, create_autotuner)

## Pre-processing

library(mlr3pipelines)

create_pipeops <- function(learner) {

po("encode") %>>% po("scale") %>>% po("learner", learner)

}

## apply our function

learners <- lapply(learners, create_pipeops)

## benchmark

## select holdout as the resampling strategy

resampling <- rsmp("cv", folds = 3)

# ## add KM and CPH

# learners <- c(learners, lrns(c("surv.kaplan", "surv.coxph")))

design <- benchmark_grid(tasks, learners, resampling)

print(design)

set.seed(123)

bm <- benchmark(design, store_models = T)

## Aggreggate with Harrell's C and Integrated Graf Score

msrs <- msrs(c("surv.cindex"))

mlr3misc::map(as.data.table(bm)$learner, "model")

library(tidyverse)

library(ggplot2)

library(ggstatsplot)

library(survival)

library(stringr)

library(viridis)

library(forestplot)

library(scales)

tcga.surv<- read.table("tcgasurv.txt", row.names = 1, check.names = F, stringsAsFactors = F, header = T,sep = "\t")

tcga.surv <- tcga.surv[,c("status","futime","Age","Gender","Stage","riskscore")]

mulcox.tcga <- summary(coxph(Surv(futime, status) ~ ., data = tcga.surv))

mulcox.tcga <- data.frame(variable = rownames(mulcox.tcga$conf.int),

HR = mulcox.tcga$conf.int[,1],

lower.95CI = mulcox.tcga$conf.int[,3],

upper.95CI = mulcox.tcga$conf.int[,4],

p = mulcox.tcga$coefficients[,5],

stringsAsFactors = F)

rownames(mulcox.tcga) <- NULL

head(mulcox.tcga)

hrtable <- rbind(c("Multivariate Cox",NA,NA,NA,NA),

c("TCGA Cohort",NA,NA,NA,NA),

mulcox.tcga)

tabletext <- cbind(c("Variable",hrtable$variable),

c("HR",format(round(as.numeric(hrtable$HR),3),nsmall = 3)),

c("lower 95%CI",format(round(as.numeric(hrtable$lower.95CI),3),nsmall = 3)),

c("upper 95%CI",format(round(as.numeric(hrtable$upper.95CI),3),nsmall = 3)),

c("pvalue",formatC(as.numeric(hrtable$p), format = "e", digits = 2)))

tabletext

nrow(tabletext) + 1

tabletext[2,] <- c("Multivariate Cox",NA,NA,NA,NA)

tabletext[3,] <- c("TCGA Cohort",NA,NA,NA,NA)

pdf("forestplot of risk table.pdf", width = 8, height = 6)

forestplot(labeltext=tabletext,

mean=c(NA,as.numeric(hrtable$HR)),#log2(HR)

lower=c(NA,as.numeric(hrtable$lower.95CI)),

upper=c(NA,as.numeric(hrtable$upper.95CI)),

graph.pos=6,

graphwidth = unit(.25,"npc"),

fn.ci_norm="fpDrawDiamondCI",#

col=fpColors(box="#00A896", lines="#02C39A", zero = "black"),

boxsize=0.4,#

lwd.ci=1,clip = c(-Inf, 6),

ci.vertices.height = 0.1,ci.vertices=F,#

zero=1,#

lwd.zero=2,#

xticks = c(0,1,2,3,4,5,6),

lwd.xaxis=2,

xlab=expression("HR"),#"log"[2]~"

hrzl_lines=list("1" = gpar(lwd=2, col="black"),

"3" = gpar(lwd=1, col="grey50", lty=2),

"13" = gpar(lwd=2, col="black")),1

txt_gp=fpTxtGp(label=gpar(cex=1.2),#

ticks=gpar(cex=0.85),

xlab=gpar(cex=1),

title=gpar(cex=1.5)),

lineheight = unit(.75,"cm"),

colgap = unit(0.3,"cm"),

mar=unit(rep(1.5, times = 4), "cm"),

new_page = F

)

invisible(dev.off())

realdata <- tcga.surv

#realdata[1:3,1:6]

Coxoutput=data.frame()

for(i in colnames(realdata[,3:ncol(realdata)])){

cox <- coxph(Surv(futime, status) ~ realdata[,i], data = realdata)

coxSummary = summary(cox)

Coxoutput=rbind(Coxoutput,cbind(gene=i,HR=coxSummary$coefficients[,"exp(coef)"],

coef=coxSummary$coefficients[,"coef"],

z=coxSummary$coefficients[,"z"],

pvalue=coxSummary$coefficients[,"Pr(>|z|)"],

lower=coxSummary$conf.int[,3],

upper=coxSummary$conf.int[,4]))

}

for(i in c(2:6)){

Coxoutput[,i] <- as.numeric(as.vector(Coxoutput[,i]))

}

Coxoutput <- arrange(Coxoutput,pvalue)# %>%

head(Coxoutput)

hrtable <- rbind(c("Univariate Cox",NA,NA,NA,NA),

Coxoutput)

tabletext <- cbind(c("Variable",hrtable$gene),

c("HR",format(round(as.numeric(hrtable$HR),3),nsmall = 3)),

c("lower 95%CI",format(round(as.numeric(hrtable$lower),3),nsmall = 3)),

c("upper 95%CI",format(round(as.numeric(hrtable$upper),3),nsmall = 3)),

c("pvalue",formatC(as.numeric(hrtable$p), format = "e", digits = 2)))

tabletext

nrow(tabletext) + 1

tabletext[2,] <- c("Univariate Cox",NA,NA,NA,NA)

pdf("Unicox.pdf", width = 8, height = 6)

forestplot(labeltext=tabletext,

mean=c(NA,as.numeric(hrtable$HR)),#log2(HR)

lower=c(NA,as.numeric(hrtable$lower)), #log2(95%)

upper=c(NA,as.numeric(hrtable$upper)),#log2(95%)

graph.pos=6

graphwidth = unit(.25,"npc")

fn.ci_norm="fpDrawDiamondCI"

col=fpColors(box="#00A896", lines="#02C39A", zero = "black")

boxsize=0.4,#box

lwd.ci=1,clip = c(-Inf, 5)

ci.vertices.height = 0.1,ci.vertices=F

zero=1,#zero

lwd.zero=2,#zero

xticks = c(0,0.5,1,1.5,2,2.5,3,3.5,4,4.5,5)

lwd.xaxis=2,

xlab=expression("HR"),#"log"[2]~"

hrzl_lines=list("1" = gpar(lwd=2, col="black")

"2" = gpar(lwd=1, col="grey50", lty=2)

#"9" = gpar(lwd=1, col="grey50", lty=2),

"15" = gpar(lwd=2, col="black"))

txt_gp=fpTxtGp(label=gpar(cex=1.2)

ticks=gpar(cex=0.85),

xlab=gpar(cex=1),

title=gpar(cex=1.5)),

lineheight = unit(.75,"cm")

colgap = unit(0.3,"cm"),

mar=unit(rep(1.5, times = 4), "cm"),

new_page = F

)

invisible(dev.off())

tr <- tcga.surv

ROC.a <- timeROC(T=tr$futime,

delta=tr$status, marker=tr$risk_score,

#other_markers=as.matrix(tr[,c("age","sex")]),

cause=1,

weighting="marginal",

times=c(0,0.5,1,1.5,2,2.5,3,3.5,4,4.5,5,5.5,6,6.5,7,7.5,8,8.5,9,9.5,10),

iid=TRUE)

ROC.b <- timeROC(T= tr$futime, delta= tr$status, marker=tr$Age,

#other_markers=as.matrix(tr[,c("age","sex")]),

cause=1,weighting="marginal",

times=c(0,0.5,1,1.5,2,2.5,3,3.5,4,4.5,5,5.5,6,6.5,7,7.5,8,8.5,9,9.5,10),

iid=TRUE)

ROC.c <- timeROC(T=tr$futime, delta=tr$status,marker=-tr$Gender,

#other_markers=as.matrix(tr[,c("age","sex")]),

cause=1,weighting="marginal",

times=c(0,0.5,1,1.5,2,2.5,3,3.5,4,4.5,5,5.5,6,6.5,7,7.5,8,8.5,9,9.5,10),

iid=TRUE)

ROC.d <- timeROC(T=tr$futime, delta=tr$status,marker=tr$Stage,

#other_markers=as.matrix(tr[,c("age","sex")]),

cause=1,weighting="marginal",

times=c(0,0.5,1,1.5,2,2.5,3,3.5,4,4.5,5,5.5,6,6.5,7,7.5,8,8.5,9,9.5,10),

iid=TRUE)

pdf("timeROC.pdf", 6, 5)

plotAUCcurve(ROC.a, conf.int=FALSE, col="red")

plotAUCcurve(ROC.b, conf.int=FALSE, col="darkblue", add=TRUE)

plotAUCcurve(ROC.c, conf.int=FALSE, col="darkgreen", add=TRUE)

plotAUCcurve(ROC.d, conf.int=FALSE, col="purple", add=TRUE)

legend("topright", c("Riskscore","Age","Gender","Stage"),

col=c("red","darkblue","darkgreen","purple"),

bty='n', lty=1, lwd=2, cex=0.8)

dev.off()

library(survival)

library(ggplotify)

library(magick)

library(cowplot)

pbc <- tcga.surv

Srv = Surv(pbc$futime, pbc$status)

coxmod1 = coxph(Srv ~Age+Stage+Subtype+, data=pbc)

pbc$model1 = c(1 - (summary(survfit(coxmod1, newdata=pbc), times=3)$surv))

coxmod2 = coxph(Srv ~ ascites + spiders + edema + chol + albumin + alk.phos + ast + trig + platelet + protime, data=pbc)

pbc$model2 = c(1 - (summary(survfit(coxmod2,newdata=pbc), times=3)$surv))

coxmod3 = coxph(Srv ~ trt + age + sex + hepato + catbili + copper + stage + ascites + spiders + edema + chol + albumin + alk.phos + ast + trig + platelet + protime, data=pbc)

pbc$model3 = c(1 - (summary(survfit(coxmod3,newdata=pbc), times=3)$surv))

coxmod4 = coxph(Srv ~ stage, data=pbc)

pbc$model4 = c(1 - (summary(survfit(coxmod4,newdata=pbc), times=3)$surv))

head(pbc)

pdf("net_benefit.pdf",width = 6,height = 6)

stdca(data=pbc, outcome="status", ttoutcome="time", timepoint=3,

predictors=c("model1","model2","model3","model4"),

cmprsk=TRUE, smooth=TRUE,

xstop=0.5,intervention="FALSE")

dev.off()

library(survival)

library(regplot)

library(rms)

library(nomogramEx)

dd <- datadist(pbc)

pbc$Stage <- factor(pbc$Stage,

labels = c("I", "II", "III", "IV"))

pbc$Gender<-factor(pbc$Gender,labels=c("Male","Female"))

options(datadist="dd")

options(na.action="na.delete")

summary(pbc$time)

coxpbc <- cph(formula = Surv(futime,status) ~ Age + Gender + Stage + Riskscore+Grade,data=pbc,x=T,y=T,surv = T,na.action=na.delete)

print(coxpbc)

surv <- Survival(coxpbc)

surv3 <- function(x) surv(1,x)

surv4 <- function(x) surv(3,x)

surv5 <- function(x) surv(5,x)

x <- nomogram(coxpbc,fun = list(surv3,surv4,surv5),lp=T,

funlabel = c('1-year survival Probability','3-year survival Probability','5-year survival Probability'),

maxscale = 100,fun.at = c(0.95,0.9,0.8,0.7,0.6,0.5,0.4,0.3,0.2,0.1))

pdf("nomogram_classical.pdf",width = 12,height = 10)

plot(x, lplabel="Linear Predictor",

xfrac=.35,varname.label=TRUE, varname.label.sep="=", ia.space=.2,

tck=NA, tcl=-0.20, lmgp=0.3,

points.label='Points', total.points.label='Total Points',

total.sep.page=FALSE,

cap.labels=FALSE,cex.var = 1.6,cex.axis = 1.05,lwd=5,

label.every = 1,col.grid = gray(c(0.7,0.8, 0.95)))

dev.off()

f1 <- cph(formula = Surv(futime,died) ~ Age+Grade+Stage+Gender+riskscore,data=pbc,x=T,y=T,surv = T,na.action=na.delete,time.inc = 1)

cal1 <- calibrate(f1, cmethod="KM", method="boot",u=1,m=100,B=1000)

f3 <- cph(formula = Surv(futime,died) ~ Age+Grade+Stage+Gender+riskscore,data=pbc,x=T,y=T,surv = T,na.action=na.delete,time.inc = 3)

cal3 <- calibrate(f3, cmethod="KM", method="boot",u=3,m=100,B=1000)

f5<- cph(formula = Surv(futime,died) ~ Age+Grade+Stage+Gender+riskscore,data=pbc,x=T,y=T,surv = T,na.action=na.delete,time.inc = 5)

cal5 <- calibrate(f5, cmethod="KM", method="boot",u=5,m=100,B=1000)

pdf("calibration_compare.pdf",width = 6,height = 6)

plot(cal1,lwd = 2,lty = 1,errbar.col = c("#8ecfc9"),

bty = "o"

xlim = c(0,1),ylim= c(0,1),

xlab = "Nomogram-prediced OS (%)",ylab = "Observed OS (%)",

col = c("#8ecfc9"),

cex.lab=1.2,cex.axis=1, cex.main=1.2, cex.sub=0.6)

lines(cal1[,c('mean.predicted',"KM")],

type = 'b', lwd = 1, col = c("#8ecfc9"), pch = 16)

mtext("")

plot(cal3,lwd = 2,lty =1,errbar.col = c("#fa7f6f"),

xlim = c(0,1),ylim= c(0,1),col = c("#fa7f6f"),add = T)

lines(cal3[,c('mean.predicted',"KM")],

type = 'b', lwd = 1, col = c("#fa7f6f"), pch = 16)

plot(cal5,lwd = 2,lty =1,errbar.col = c("#82b0d2"),

xlim = c(0,1),ylim= c(0,1),col = c("#82b0d2"),add = T)

lines(cal5[,c('mean.predicted',"KM")],

type = 'b', lwd = 1, col = c("#82b0d2"), pch = 16)

abline(0,1, lwd = 2, lty = 3, col = c("#224444"))

legend("topleft"

legend = c("1-year","3-year","5-year"), #

col =c("#8ecfc9","#fa7f6f","#82b0d2"), #

lwd = 2,#

cex = 1.2,#

bty = "n")

dev.off()
